# Place your bet on change: the Young Adult Action Collective leading community-academic partnership efforts to understand and address gambling harms in Springfield, MA

**DOI:** 10.3389/fpubh.2026.1736067

**Published:** 2026-05-07

**Authors:** Geraldine Puerto, Theresa Glenn, Linnea A. Evans, Suraya Arnold, Brenda D. Evans, Blu Figueroa, Tayy Floyd, Tykie Greene, Victor Martinez, Monet Murphy, Rosemarie Oliveras, Jaydynn Ramos, James Rosado, Gigi Sanchez, Jarix Santiago, Rachel A. Volberg, Kathryn P. Derose

**Affiliations:** 1 Department of Health Promotion and Policy, School of Public Health and Health Sciences, University of Massachusetts Amherst, Amherst, MA, United States; 2 Office of Problem Gambling and Prevention, Department of Health & Human Services, Springfield, MA, United States; 3 Young Adult Action Collective, The Gambling Awareness Research Initiative, Springfield, MA, United States; 4 Youth Services Center, New North Citizens’ Council, Springfield, MA, United States; 5 Department of Biostatistics and Epidemiology, School of Public Health and Health Sciences, University of Massachusetts Amherst, Amherst, MA, United States

**Keywords:** crowdsourcing contest, digital storytelling, gambling harm, participatory action research, young adults

## Abstract

This community case study describes the development and implementation of the Gambling Awareness Research Initiative (GARI), a community-academic research partnership in Springfield, Massachusetts, created in response to the expansion of legalized gambling and the need for community-driven research to further understand gambling’s impact on youth and young adults. The initiative uses a participatory action research design that positions the Young Adult Action Collective, a group of young adults from the city, as co-researchers and co-leaders, working in partnership with the city’s Department of Health & Human Services, a youth-serving community organization, and a university research team. Methodologically, GARI explores the adoption of arts-based research, such as digital storytelling and a creative crowdsourcing contest, as potential approaches to illuminate a stigmatized and often hidden public health issue like gambling. The paper highlights the process elements that make this model effective, including radical love, collective learning and growth, and young-adult-driven research development, as well as the logistical and structural challenges that arise from balancing research priorities and the realities of young-adult participation. Community work is, at its core, about building relationships and doing work that endures past the project at hand. Through this process, something meaningful is created: a kind of knowledge and connection that has the power to transform individuals, groups, and communities over time. As the public health field continues to confront complex health and social issues, GARI exemplifies how community-driven, arts-based methodologies can help engage young adults in shaping both the research questions and the pathways toward change. Such approaches hold the potential to deepen understanding, challenge extractive research norms, and strengthen the connections between the community and academia.

## Introduction

Gambling-related harms are a growing public health concern, particularly among young adults who are navigating early financial independence, social transitions, and exposure to digital forms of gambling (1, 2). In Massachusetts, the expansion of legalized gaming has created new social and environmental contexts that may shape local attitudes and behaviors toward gambling. These contexts are further affected by more commercial determinants of health, as the gambling industry increasingly shapes exposure through marketing, product design, and digital access (3). Since the opening of the MGM Resort Casino in Springfield, Massachusetts in 2018, participation in gambling has risen and statewide surveys show that 1.4% of adults meet criteria for “problem gambling” and another 8.5% are at risk (4). Young adults—especially those facing economic or educational disadvantage—appear disproportionately vulnerable (4, 5).

Springfield, Massachusetts, is a critical setting to study these dynamics. The city’s 153,000 residents represent broad racial and ethnic diversity—approximately 47% Latino, 19% Black or African American, and 27% White—with nearly a quarter between the ages of 15 and 29 (6). Economic inequities are pronounced: almost 30% of residents live below the federal poverty line, and the median household income of $47,101 is less than the state median (6). Limited socio-economic opportunities, together with proximity to local and regional casinos and widespread online sports betting, create conditions that may intensify gambling exposure among young adults seeking connection or financial relief. In fact, recent research found that 64% of young adults in Springfield ages 18–34 reported gambling in the last year (7). This figure is similar to national data, which show that 74% of 18–24-year-olds gambled in the past year (8). Further, national data trends indicate that the median number of gambling activities in the past year among these youngest gamblers (18 to 24 years old) has gone from 4 in 2018 to 8 in 2021 (8).

Current research on gambling harm among U.S. young adults is limited and fragmented. Studies link early gambling onset to later mental health and substance-use problems (9, 10) and note emerging risks tied to newer forms such as micro-betting and loot boxes (11, 12). Yet few investigations consider how contextual factors—including local environments, family influence, and digital accessibility—shape vulnerability and resilience (13, 14). This gap underscores the need for community-based, participatory research to better understand the social dimensions of gambling harm among young adults. Further, community-specific qualitative data and participatory research strategies can provide contextual insight into the “how” and “why” of gambling by engaging affected community members in defining the problem and building solutions. In response, the Gambling Awareness Research Initiative (GARI) was launched in 2023 through a partnership among the Springfield Department of Health and Human Services (SDHHS), New North Citizens’ Council (NNCC), and the University of Massachusetts Amherst (UMass) Center for Community Health Equity Research. To date, GARI has been funded by the Massachusetts Gaming Commission’s Community Mitigation Fund and engages Springfield young adults as co-researchers to examine how gambling intersects with social determinants and its impacts on families and communities.

GARI employs participatory and arts-based research methods—including digital storytelling, the *Wagers & Whispers* creative crowdsourcing contest, and community forums—to promote dialogue, co-create knowledge around gambling harms, and generate culturally grounded prevention and mitigation strategies. The combination of participatory and arts-based methods helps participants express complex, stigmatized, or difficult-to-articulate experiences in ways that traditional research methods may not capture, while fostering trust, reflection, and collective dialogue (15–20). Rather than view gambling solely as an individual behavior, GARI situates it within broader social and economic systems and seeks to reimagine prevention through community ownership and youth voice. We recognize ongoing discussions in the field about the use of stigmatizing language and how gambling-related impacts are defined, including distinctions between harms experienced by individuals who gamble and those affecting others around them (3). We use the term *gambling harms* here to reflect a broader framing that extends beyond the person experiencing gambling problems to include impacts on family, friends, other social networks, and communities.

This community case study documents the development and implementation of GARI, highlighting how equitable, young adult-led partnerships can inform public health interventions and advance research on gambling harm prevention in economically marginalized urban settings. Future publications will report research findings from GARI, but here we focus on the *process* of developing and implementing this young-adult led partnership to understand and address gambling harm. There is limited guidance in the literature on how to operationalize partnerships with young adults around gambling-related harm, particularly within racially and ethnically diverse, economically marginalized urban settings. Our in-depth description in this article about *how* the partnership developed, *what* it did, and the *lessons learned in the process* can inform others wishing to work with young adults and other key populations to understand and address gambling-related harm.

## Context

### The approach

GARI used Participatory Action Research, with young adult as co-researchers to identify issues, collect and analyze data, and lead action to address problems in their communities (21, 22). Through PAR, GARI centers the voices and lived experiences of young adults of color, who are often excluded from dictating the research and action process (23). By actively engaging young adults as co-researchers, the method not only generates rich, authentic insights about gambling and its impacts in Springfield but also builds capacity among its members to take ownership of solutions. This approach fosters leadership, critical thinking, and community advocacy among the young adults participating, while ensuring that the project’s outcomes reflect the realities and priorities of those most affected. For GARI, PAR was seen as a means to enhance both the credibility and relevance of future project findings, making them more actionable for local policy, prevention, and awareness efforts.

Similar to the “7Cs of Participatory Action Research” framework (24), our PAR approach incorporated several intentional elements during the planning and implementation of the research to operationalize participatory principles in practice. We engaged in a cyclical process of analyzing gambling harms across phases, continually adapting our approach based on emerging insights and the rapidly evolving gambling landscape. We emphasized collaborative conversations about what it meant for YAAC members to serve as co-researchers, consistently considered the contexts in which research and outreach activities were taking place, and intentionally bridged theory and practice when making decisions. Across phases, we created space to recap and reflect on conversations, note patterns and shifts over time, clarify roles, and revisit shared goals, while prioritizing ongoing relationship-building through dialogue and collective activities.

**Figure 1:**
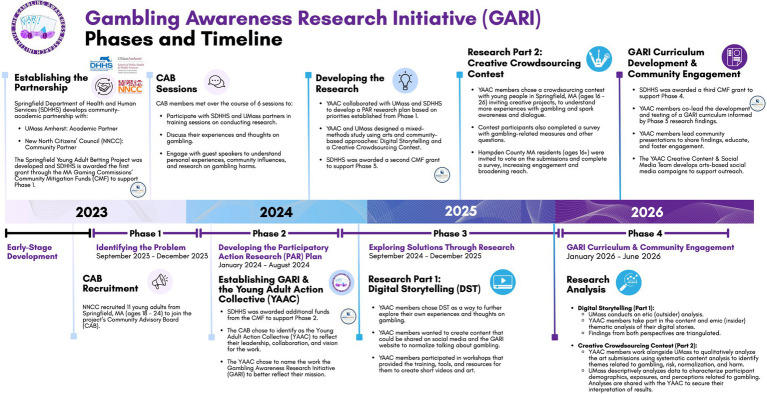
Gambling awareness research initiative (GARI) phases and timeline.

Figure 1 provides an overview of the process and timeline of GARI from 2023 to 2026, which can be divided into four distinct phases. We elaborate on each of the phases, primary activities, and milestones below.

### Establishing the partnership

As noted above, three institutional partners came together to launch the initiative, following key principles of community-based participatory research (CBPR)—equity, shared power, community leadership, mutual trust, and co-created knowledge (25–29). SDHHS has been the overall lead and recipient of the grant funding for GARI and has subcontracted with UMass and NNCC. The three institutional partners have met regularly (weekly or biweekly) to develop and implement the plan for engaging young adults in the work. Additionally, NNCC (RO, JS) has played a strong role in recruiting young adults to participate in the project and has hosted the meetings with young adults, with SDHHS (TG) and UMass (mostly GP, with mentorship and support from LE, BE, RV, KD) co-facilitating the meetings.

In conceptualizing the work with the young adults, the partners drew on Adrienne Maree Brown’s facilitation philosophy to emphasize “holding space,” to create an environment for non-extractive, community-led research. Brown describes this as supporting groups to collaborate and navigate conflict in ways that are accountable and generative (30). Guided by these values, partners co-developed community agreements with the young adults defining how we would work together, including meeting structures, decision-making, and conflict resolution, emphasizing transparency and “moving at the speed of trust” (30). We recognized that sustaining engagement relied on strong relationships, built through personal connection, play, and responsiveness.

Beyond meeting research aims on addressing gambling harms and public health, the initiative has sought to offer growth and skill-building opportunities for the young adult co-researchers. Tasks were aligned with young adult co-researchers’ interests—from project management and outreach to design, communications, and research—supporting both collective and individual development.

## Key programmatic elements

Below we outline the primary components of GARI and its work from 2023 to 2026. In doing so, we write as a collective representing diverse experiences, roles, and perspectives—academic, public health, community-based organizational, and emerging young adult leadership—all of whom serve as co-authors. Throughout the manuscript, we distinguish when particular ideas or reflections originate from the young adult co-authors, referring to the Young Adult Action Collective (YAAC), as described below, recognizing their lived experience as central to the interpretation and direction of this work. Because these young adults are co-researchers and co-authors rather than study participants, their voices come through shared narrative rather than through quotations, ensuring that their analysis and perspectives are woven directly into the collective account.

### The young adult action collective

#### Recruitment and participation

A key component of GARI was the formation of the Young Adult Action Collective (YAAC). Recruitment took place over several phases, each shaped by the initiative’s evolving goals, available resources, and lessons from earlier stages.

Phase 1 recruitment was led by NNCC and focused on young adults ages 18 to 24 who were available to participate in a six-week community advisory board. This timeline was shaped by the scope of the initial funding opportunity, which supported a pilot phase aimed at forming the advisory board and identifying research priorities from the perspectives of young adults in Springfield. Recruitment messaging emphasized willingness to share personal knowledge, insights, and experiences related to gambling and its community-level impacts, and encouraged diverse candidates to apply. The application included questions exploring applicants’ motivations for joining, their leadership and community involvement, and relevant personal or professional experience. Additionally, applicants were asked to share information about their gender identity, race, and ethnicity. All the questions reflected the team’s commitment to building a cohort that represented diverse voices and perspectives. The team aimed to recruit 10 young adults—an intentional choice to balance diverse perspectives with group cohesion and trust. Nineteen applications were received, and NNCC conducted interviews. In the end, 10 young adults were selected by NNCC as the best fit for the program’s goals according to the recruitment criteria.

Phase 1 was designed as a formative, time-limited engagement. The structured six-week format worked well for participants at that stage, but when the project was able to obtain additional funding to continue the process and design and implement a PAR agenda (Phase 2 and 3), expectations shifted to an ongoing commitment of at least one year, with recurring meetings and responsibilities. This change did not work for everyone, given competing demands like school and work. It is worth noting, however, that members who chose to continue have remained with the project since, reflecting meaningful retention among those for whom the longer-term commitment was feasible.

Phase 3 recruitment was co-led by NNCC, SDHHS, and existing YAAC members. The application process mirrored Phase 1. However, YAAC members approached this phase with particular intentionality, recognizing the importance of protecting the values and energy that had become central to the work and team dynamics. They wanted to purposively recruit young adults whose perspectives and presence aligned with the group’s shared commitments, and the YACC played an active role by recruiting at community events, reaching out through personal networks, and participating in applicant interviews. In addition to age eligibility, recruitment in this phase emphasized a desire to address gambling-related impacts in the community, a demonstrated passion for making a difference, and an artistic passion aligned with the project’s creative components. Availability for biweekly meetings, with optional remote work hours, was also required. Fourteen applications were received, and six young adults were selected.

Eligibility criteria remained deliberately broad; applicants were not required to have personal gambling experience. Instead, recruitment materials emphasized openness to discuss gambling and its community impacts. This approach welcomed varied lived experiences, from direct and indirect exposure to limited familiarity. Group discussions later expanded members’ shared understanding of gambling, including activities such as gaming-related monetization. Additionally, we incorporated selection criteria during the review of applications to intentionally build a cohort diverse across race, ethnicity, age, gender, and sexuality.

YAAC members were paid for all phases, with rates adjusted in response to funding and member feedback. In Phase 1: $100 per session for six 2-hour sessions. In Phase 2: $100 per 2.5 h (including meals/check-in) and $50/h for remote work, which was limited and structured to specific tasks and time allocations. Phase 3: After budgeting discussions, compensation shifted to $100 per in-person meeting and $25/h for remote work. Phase 4: Following further funding cuts, a flat $25/h rate was adopted. While YAAC members expressed concern about reduced pay, they remained committed to the initiative and engaged in transparent dialogue about structural funding challenges—highlighting common tensions in participatory research between equitable compensation and financial limitations.

The YAAC now includes nine Springfield, MA residents aged 18–26, representing diverse genders, LGBTQIA+ identities, and Black/African American, Hispanic/Latine, and multiracial backgrounds. Members bring a range of relationships to gambling—from personal participation and family exposure to critical perspectives informed by community impacts.

#### The importance of lived experience

Originally, the young adult members participated as a community advisory board (CAB) but soon recognized the collective strength as co-leaders with other partners. As their involvement deepened, they began shaping research questions, outreach methods, and decisions about community engagement rather than simply advising about them. They realized that real understanding of gambling and its social impacts comes from lived experience—the kind that they, as community members, possess. They shared experiences that often overlapped with the very harms the initiative aimed to address. Several noted how financial strain, housing instability, and emotional exhaustion blurred the line between studying gambling harm and being vulnerable to it. When basic supports like healthcare, employment, or shelter felt inaccessible, the around-the-clock availability of casinos or other gambling spaces appeared as one of few available options—highlighting how extractive systems can be more accessible than systems of care.

Work environments added further complexity. One member whose past employment involved selling gambling products at her job described being embedded within the system, watching customers spend entire paychecks on lottery tickets while feeling complicit in the process. Such observations revealed how economic desperation and hope for something more can be a primary determinant of gambling and shaped members’ understanding of it as both personal and structural. For many, gambling symbolized a shortcut to stability or success that otherwise felt out of reach – home ownership, healthcare, or entrepreneurship. Members who had experienced frequent moves or housing insecurity expressed how the hope of a “big win” represented the dream of stability. Members also discussed their own gambling behaviors, such as sports betting, acknowledging moments when confidence in skill blurred into risk-taking.

From that perspective, the group affirmed that the initiative’s power lies in its “for the people, by the people” approach, ensuring that those who are often most targeted lead the conversation and guide the work forward. In response, the group chose to identify as the Young Adult Action Collective (YAAC) to more accurately reflect their leadership, collaboration, and vision for the work. YAAC members describe the collective as a group of young adults who come together with a shared mission to heal their community through action that fosters awareness and change. For the YAAC, a central focus of this initiative is how both the process and outcomes can benefit the broader community. When reflecting on what community means to them, YAAC members emphasized that while the initiative is rooted in Springfield, MA, they believe its purpose extends beyond place and is intended to contribute to society more broadly, but especially racially and ethnically minoritized and economically disinvested communities. The collective emphasizes its shared passion for preventing gambling harms and addressing its misconceptions—particularly the tendency to overlook how chance-based mechanisms are embedded in our experiences from very early ages to normalize gambling.

#### YAAC operations and reflections

The YAAC meets weekly with facilitators from the SDHHS and UMass to work on the ongoing research and program tasks, collaborate with one another, and stay informed about developments related to gambling and broader social and political issues. Each meeting begins with dinner and personal check-ins, creating space to support one another before moving into the work. Meeting agendas and research activities are shaped collaboratively by all partners, with the YAAC leading specific work forward, addressing challenges, planning next steps, and discussing both successes and lessons learned along the way. Work outside the regular YAAC weekly meetings is carried out by members whose availability, interests, and strengths align with specific activities or areas they are eager to develop. In addition, one YAAC member serves in a more hands-on, part-time role, working closely with the project lead at SDHHS and representing the collective at the biweekly full-partner meetings involving SDHHS, UMass, and NNCC. This structure ensures that YAAC voices remain central to all aspects of the project, even when not all members can participate outside of regular weekly meetings.

In reflecting on what distinguishes the YAAC from other youth initiatives, members highlight their unique position as both researchers and community insiders. They note that their work blurs the boundary between “being studied” and “doing the studying,” enabling them to explore gambling as an issue affecting their own lives and communities. Additionally, the group strongly believes that GARI has fostered an inclusive space for young people to get involved, regardless of background, age, race, sexuality, or gender. The group’s diversity emerged as one of its greatest strengths. Members described how their different backgrounds and experiences were a constant source of learning, expanding how they see the world and each other. The group also spoke about challenging stereotypes often associated with low-income, racialized, and minoritized communities, underscoring their commitment to demonstrating care, resilience, and agency. Overall, there is a shared sense of pride in demonstrating that young people are aware of the issues affecting their city and are deeply committed to addressing them as emerging leaders in public health research and community initiatives.

Members of the YAAC describe feeling a deep sense of purpose and fulfillment in their roles as co-researchers. They share that being part of this project gives them a meaningful way to look deeper into themselves and their community while contributing to something larger. The process feels engaging and welcoming—a unique opportunity to learn, create, and connect without pressure. As co-researchers and co-leaders, they believe they give proper consideration and care to the people the project aims to reach. Together, they express that contributing to the collective knowledge around gambling and public health, especially for their own city, feels empowering and positive.

### The phases of GARI’S work

GARI’s work to date has occurred across three phases, as depicted in Figure 1: Phase 1: Identifying the Problem; Phase 2: Developing the PAR Plan; Phase 3: Exploring Solutions through Research; Phase 4: GARI Curriculum & Community Engagement. During Phase 1, YAAC members participated with SDHHS and UMass partners in training sessions on conducting research, engaged in discussions about their experiences and thoughts on gambling, and listened to guest speakers to understand personal experiences, community influences, and research on gambling harms. Building on Phase 1, the YAAC collaborated with UMass researchers and SDHHS public health professionals to develop a PAR plan during Phase 2 of the project. This plan was developed based on methodologies that the YAAC deemed most effective for engaging youth and young adults, such as arts and community-based approaches. During Phase 3, the team (UMass, YAAC, SDHHS, NNCC) implemented the Digital Storytelling and Creative Crowdsource Contest. In Phase 4 (in progress), the YAAC is leading the translation of research into action by developing a research-informed community engagement plan and educational curriculum to implement in the city of Springfield and neighboring communities.

### Research planning and development

During the research planning phases (Phases 1 and 2), bi-directional knowledge sharing and co-learning were central. Through educational sessions on gambling, related harms, and their connections to the social determinants of health, we engaged in group discussions about how gambling risks show up in the lives of the YAAC members and their communities. Together, we explored how gambling affects young adults and deepened both our collective and individual understandings of the underlying factors that can lead to gambling harms. The insider perspective of the YAAC has become a defining feature of the project. As members began to see themselves and their communities reflected in the data and in conference presentations, they recognized that GARI’s work stands apart from much of the existing research, which is often conducted from an outside perspective. Their lived experience brings an internal understanding that adds depth, context, and meaning to what is being studied. At the close of Phase 1, the YAAC ultimately identified priority themes that could inform future research, including gambling as a social phenomenon, high prevalence of misconceptions of gambling, the lack of gambling literacy among youth and young adults, and the need for early education to reduce risks among youth and young adults (see Table 1).

**Table 1:** Gambling-related themes identified by YAAC members during phase 1.

Theme	Description
Gambling as a social phenomenon	Through conversation, YAAC members emphasized the social dimensions of gambling among young adults, highlighting its critical role in shaping gambling behavior. “Gentlemen’s bets,” truth or dare, online gaming, and arcade activities represent some of the ways youth begin engaging with gambling-adjacent activities from a young age. Discussions also explored motivations for engagement, including non-monetary influences such as competition, status, peer pressure, ego, and pride. Belonging to social groups like sports teams and online gaming communities was identified as contributing to increased gambling exposure and experimentation.
Gambling misconceptions and the importance of gambling literacy among young adults and adolescents	Many YAAC members reported early exposure to gambling, including receiving lottery tickets for gifts as young as age six and being asked to purchase tickets for adult family members. Exposure commonly occurred through family and media with little to no education about gambling literacy or related harms. This was especially true for video gaming, where in-game gambling features often go unrecognized as gambling—as one YAAC member reflected, many people are unaware that what they are doing is, in fact, gambling.
Determining if specific gambling literacy education could lower the risk of problem gambling among young adults and adolescents	YAAC members agreed that gambling literacy education is critically needed for peers and middle and high-school-age youth—particularly younger adolescents with less gambling exposure—to reduce the future incidence of problem gambling. They also emphasized that pairing gambling literacy with financial literacy would help young people manage personal budgets, distinguishing discretionary from essential spending, and develop saving habits for unexpected needs.
Ways that the transition period from adolescence to young adulthood may increase young adults’ vulnerability to problem gambling	YAAC members discussed how social, economic, and other issues may increase the risk of problem gambling among young adults—for example, the challenges young adults often face while striving for financial and emotional independence, including difficulties managing financial resources and the lack of social support.

During Phase 2, we reviewed findings from previous gambling studies and examined the research methods used in both gambling research and PAR to inform how we would approach our own work. During this phase, we refined our study’s focus areas and explored a range of research methods, including surveys, focus groups, photovoice, digital storytelling, zines, and creative crowdsourcing contests. YAAC members were most drawn to arts-based and community-centered approaches because these aligned with their own experiences and interests in creative expression as powerful ways to tell stories and share lived experiences. They believed that by exploring gambling through artistic mediums themselves, and by inviting other youth and young adults in the city to share their own stories in the medium of their choice, they could gain a deeper and more diverse understanding of how gambling affects their communities. From this vision, the idea to combine two arts-based participatory methods —digital storytelling and a creative crowdsourcing contest—was born.

### GARI’S current two-part research process

GARI’s current research integrates two complementary components: digital storytelling (Part 1) and a creative crowdsourcing contest (Part 2). These methods were intentionally combined to center both personal and community perspectives.

Part 1 focuses on the YAAC members’ own experiences, positioning them as both the subjects and co-researchers of the inquiry. Through digital storytelling, YAAC members explored their personal and collective relationships with gambling. Building on this foundation, Part 2 engaged the YAAC more deeply as co-researchers, expanding the research to gather broader community insights into youth and young adults’ perceptions of gambling, their personal experiences, and their ideas for prevention and intervention.

Table 2 provides an overview of these two research components, identifying primary research aims and questions, phases, and roles of the various partners.

**Table 2:** Design and description of the gambling awareness research initiative (GARI) phase 3 research: part 1 and part 2.

	Research part 1: digital storytelling	Research part 2: creative crowdsourcing contest
Research questions	From the perspective of older adolescents and young adults, what counts as problematic gambling? How does gambling affect the health and wellbeing of older adolescents and young adults, and their family, friends, and communities?	What are older adolescents’ and young adults’ perspectives on arts-based prevention and treatment messaging for problem gambling? What is the extent of youth and young adults’ exposure to risk factors associated with problem gambling behavior? What are youth and young adults’ perceptions of (a) what counts gambling, (b) who is at risk for problem gambling, and (c) effective strategies to prevent and reduce problem gambling?
Aims	Part 1 focused on the Young Adult Action Collective (YAAC) members’ own experiences, positioning them as both the subjects and co-researchers of the inquiry. Through digital storytelling, YAAC members aimed to explore their personal and collective relationships with gambling, curious about what new insights might emerge through the process of digital storytelling.	Part 2 engaged the Young Adult Action Collective (YAAC) more deeply as co-researchers, expanding the research to gather broader community insights into youth and young adults’ perceptions of gambling, their personal experiences, and their ideas for prevention and intervention.
Steps	*Step 1:* Development of digital storytelling workshop sessions *Step 2:* Orientation of digital storytelling process with the YAAC *Step 3:* Group brainstorming sessions to explore the topic of gambling, as a way to generate ideas for their digital story creation *Step 4:* Story development and group story circles *Step 5:* Development, production, and editing of digital stories through both individual and collective work *Step 6:* Collaborative thematic analysis of digital stories *Step 7:* Research dissemination	*Step 1:* Development of contest concept, contest categories and guidelines, GARI website, and data collection tools. *Step 2:* Implementation of outreach plan, community art pop-ups, and community engagement. *Step 3:* Collection of contest submissions and survey data from contest participants. *Step 4:* Implementation of community vote outreach plan, collection of community votes and survey data from voters, and internal submission scoring *Step 5:* Contest winner announcements and community celebration with an art exhibit. *Step 6:* Research dissemination and continued art exhibits around Springfield, MA and other locations.
Team roles	SDHHS: Led administrative tasks for grant Co-led the research design, workshop development, data collection, and data analysis Co-facilitated the workshop sessions and provided coaching, technical support, and continued assistance throughout the entire process NNCC: Supported YAAC recruitment and engagement Provided administrative support Provided workshop session materials and space UMass Amherst: Co-led the research design, workshop development, data collection, and data analysis Co-facilitated the workshop sessions and provided coaching, technical support, and continued assistance throughout the entire process YAAC: Research Participants Co-developed creative activities as part of workshop sessions Designed prompts for digital storytelling creation Supported one another through digital storytelling creation Co-led thematic analysis of digital stories	SDHHS: Led administrative tasks for grant Co-led the research design, contest development, data collection, and data analysis Co-led community engagement and outreach strategic planning and implementation Co-led implementation of all research activities NNCC: Supported YAAC engagement Provided administrative support Provided meeting space UMass Amherst: Co-led the research design, contest development, data collection, and data analysis Co-led implementation of all research activities Co-led community engagement and outreach strategic planning YAAC: Co-led the research design, contest development, data collection, and data analysis Designed contest categories and all contest material Developed and designed the GARI website Co-led community engagement and outreach strategic planning and implementation Co-led implementation of all research activities Scored contest submissions Developed the agenda and structure for the community celebration event

In Phase 3 of GARI, findings from both parts of the research are shared in community-based forums where key stakeholders are invited to engage with the digital stories and contest submissions. Aggregated survey results will also be presented to guide local strategies for preventing and addressing problem gambling. The structure of the two research components is described in more detail below.

#### Research part 1: digital storytelling

The first research method selected by the YAAC was digital storytelling. Also known as cellphilming, digital storytelling is an arts-based research approach in which participant-researchers create short films using their mobile devices in response to specific prompts around an area of focus (16, 31, 32). This process draws on participants’ existing expertise in observing and analyzing their everyday lives. Throughout the production process, participants engage in both group discussions and individual reflection to explore personal and social experiences, make meaning of their stories, and connect them to broader community and societal issues. In doing so, their individual narratives are transformed into collective stories that can inform and advance public health efforts rooted in social justice (16).

The YAAC wanted to use this research method to explore their own experiences and narratives, curious about what new insights might emerge through the process of digital storytelling. They understood that this approach meant they would also become “participants” in the research. During pre-workshop time, we reviewed the consent form together, using it as an opportunity to discuss how consent functions in research and why it matters—particularly as they prepared to transition into the crowdsourced contest as researchers recruiting other youth and young adults. Two YAAC members created and performed a skit explaining the components of informed consent and what it means to volunteer in research. Partners reiterated that participation was voluntary and that members could opt out without consequence, and members retained agency over whether and how their digital stories would be shared publicly, including setting conditions for dissemination.

In 2025, nine YAAC members created digital stories reflecting on their personal experiences with gambling and co-developed a ten-session workshop series grounded in the StoryCenter approach (31). Workshop activities included scriptwriting, story circles, media production, and creative exercises. Upon completion of the workshop, the academic research team conducts an etic (outsider) data analysis. Then, as part of the PAR approach, YAAC members take part in the content and emic (insider) thematic analysis of their digital stories, collaboratively identifying key themes, quotes, and messages within and across stories. Lastly, findings from both perspectives are triangulated to deepen the understanding of youth and young adult experiences with gambling. This process combines deductive and inductive approaches, drawing on methods adapted from Flicker et al. (33) and Valdez et al. (34), both of which have been used in youth participatory action research (YPAR).

#### Research part 2: creative crowdsourcing contest

The second research methodology selected by the YAAC was a creative crowdsourcing contest, called *Wagers and Whispers*. Crowdsourcing is a participatory research method for problem solving, which involves an organization having a large group attempt to solve a problem and develop solutions (35–37). Multiple randomized controlled trials have provided evidence of crowdsourcing challenge contests as an effective participatory approach to addressing health issues, where an organization or group issues an open call to the public for new ideas for addressing a particular health issue (37). For example, this approach has been used to increase community engagement in addressing food deserts (38); increasing youth involvement in COVID-19 prevention and mitigation efforts (39); and addressing medical mistrust and sexual health (37, 40) through locally defined promotion messages.

In our research, we utilized a challenge contest as one crowdsourcing method to expand the collection of data beyond the YAAC members and engage the broader community to assess exposure to and perceptions of the causes and consequences of gambling harms among older adolescents and young adults. Power-sharing occurs via community-based thinking, as opposed to the typical model where solutions are concentrated among a subset of academically trained ‘experts.’

The focus of the GARI creative crowdsourcing contest was to solicit arts-based prevention and treatment messaging around gambling harms, created by contestants via a variety of multimedia modalities (e.g., poetry, illustrations, animations, videos). Following the GARI challenge contest, individuals aged 16 and older from Springfield and Hampden County were invited to participate in a community vote to select the winners. This included members of the community at large as well as local public health professionals. Based on criteria shared on the GARI website, the art submitted was evaluated based on uniqueness, thematic relevance to gambling, clarity, emotional elicitation, and community representation and inclusivity. In addition, survey data was collected from contest participants as well as community voters, to assess (1) exposure to risk factors that increase gambling harms and (2) perceptions of what counts as gambling, who is at risk for gambling harms, and views on strategies to prevent and reduce gambling harms among youth and young adults. Online consent (and assent with guardian consent for individuals aged 16–17) was securely collected through Qualtrics for both contest entry and community voting. Contestant identifiers were removed during voting to protect anonymity. Following the contest, participants were contacted to confirm their preferences for how their artwork would be displayed publicly, including name attribution and inclusion of their motivation statement.

The design and development of the contest were led by the YAAC, with health department staff and academic researchers serving in an advisory capacity. The YAAC led in creating contest prompts to inspire areas of exploration for the submissions, adapting contest guidelines to the content area, and participating in outreach efforts to encourage submissions. Outreach efforts included writing press releases, creating social media content, writing and recording public service announcements for local radio stations, being interviewed by a radio show host and journalists from local media outlets, and leading art pop-up events at local high schools and community-based organizations. YAAC members were also active leaders in scoring submissions as part of the finalist selection process.

The crowdsourced challenge contest is currently in its final stage of analysis, and YAAC members remain co-leaders in a mixed-method analysis and dissemination of findings. First, the academic research team descriptively analyzes survey data collected alongside contest submission and community voting to characterize participant demographics, exposures, and perceptions related to gambling. The YAAC will review the analyses to determine and share as a group their interpretation of results. This will inform the presentation of findings. Second, YAAC members work alongside the academic research team to qualitatively analyze the art submissions using systematic content analysis, coding visual and textual elements to identify themes related to gambling, risk, normalization, and harm. Together, the YAAC and the research team develop presentations of these results and disseminate findings via community events and materials production, both locally and across the state.

### Phase 4: research knowledge translation

Through ongoing dialogue and creative projects, YAAC members have identified opportunities to expand the scope of their research and deepen community connections. This includes extending outreach to populations beyond those directly involved in Phase 3 of the initiative and fostering broader awareness and critical conversations about gambling harms and misconceptions. The process involves translating research findings from the digital storytelling and creative crowdsourcing contest into actionable insights that inform policies, programs, education, and services. Specifically, GARI is developing an interactive gambling harm reduction and prevention curriculum, implementing it with various groups to evaluate it, participating in community events, and developing social media campaigns to support outreach.

## Discussion

Our work with GARI underscores the importance of centering community voices in public health research and intervention design. It demonstrates the capacity of young adults as co-researchers, illustrating how equitable, community-driven approaches can deepen understanding and prevention of gambling-related harm. Over two years, we encountered both successes and challenges that reveal the complexity of community–academic partnerships, as well as the broader challenges of addressing issues shaped by larger economic and commercial systems that influence gambling promotion, perceptions, and participation.

### The commercial determinants of health and gambling harm

While GARI centers community voices and local realities, these experiences unfold within a broader commercial environment that shapes gambling exposure and harm. As noted by the *Lancet Public Health Commission on Gambling*, of which one of the co-authors (RV) is a Commissioner, (1) commercial gambling is a rapidly growing global industry and is becoming increasingly digital; (2) the harms to health and wellbeing that result from gambling are more substantial than previously understood and affect many people in addition to those who gamble; (3) the evolution of the gambling industry is at a crucial juncture and decisive action is needed to prevent and mitigate widespread harm to population health and wellbeing; and (4) stronger policy and regulatory controls focused on harm prevention and protection of public health, independent of industry or other competing influences, are needed (3).

In Massachusetts, the state’s oversight structure offers one example of such independence. The Community Mitigation Fund (CMF)—which supported GARI—was created by statute and distributes resources through a peer-review process overseen by the Massachusetts Gaming Commission. The gambling industry has no role in directing CMF-funded research, as the funds derive from mandated tax allocations rather than voluntary contributions. Safeguards like these enable projects such as GARI to remain focused on community wellbeing, unconstrained by commercial influence. Situating GARI within this global conversation reminds us that local action and care are inseparable from the structural forces that shape gambling and its related harms.

### Holding change with radical love

GARI is grounded in relationship-building and enduring commitment. Beyond funding or formal outputs, community work produces lived knowledge that strengthens individuals and collective capacity. We encourage practitioners to view communities not through deficit but as sites of possibility and power.

In his presentation titled *Radical Love as a Public Health Initiative*, Hayman (41) describes radical love as a rooted, foundational love—one capable of transforming the nature of society, much like the love found in the Beloved Community framework advanced by Dr. Martin Luther King Jr. and Grace Lee Boggs (42). Public health and organizing efforts have applied this construct to promote healing, trust, and shared responsibility across diverse contexts (43, 44).

Within GARI, YAAC members identified love as both method and motivation: caring for themselves, one another, their city, and the social justice issues shaping their lives. This collective care fueled persistence, compassion, and purpose, enabling the group to navigate challenges and sustain commitment to public health. For the collective, love is not only an emotion but an organizing principle energizing their movement toward community wellbeing. Additionally, the project required ongoing navigation of accountability, care, and expectations, grounded in radical love—i.e., reaffirming participatory commitments through actions, resisting micro-management, and allowing space for uncertainty and shared decision-making. Rather than adopting punitive responses to challenges with engagement, the team worked toward an approach that emphasized learning, reflection, and collective problem-solving, while still maintaining shared responsibility for the work.

Together, these reflections affirm that radical love functions as a practice and methodology, enabling participatory research to move beyond data collection into collective healing, shared vision, and transformation.

### Personal and collective growth among the YAAC

One goal of GARI was to support YAAC members’ personal and professional development. After 2 years, members reflected on their growth and evolution as a collective. Participation fostered professionalism, confidence, and creative expression. Several noted improvements in public speaking and leadership, while others found renewed purpose in skills they had once viewed as hobbies. One member described learning how to present with confidence in any setting and to recognize strengths they had not seen before. Another shared how what began simply as an opportunity became something transformative, rekindling creativity and motivation.

Furthermore, YAAC members actively sought and participated in professional development opportunities. This included speaking at and/or attending local and national gambling and public health conferences and events, such as the Massachusetts Gaming Commission’s 2024 Research Conference, the 2024 Massachusetts Problem Gambling Conference, Springfield’s Public Health Month Kick-Off event, and the American Public Health Association 2025 Annual Meeting. Informal narratives shared reflected changes in confidence, professional identity, and the importance of the work. While anecdotal, these experiences point to meaningful shifts in how YAAC members understood themselves including personal and collective expertise and how their voices and efforts were received across spaces.

Overall, the YAAC valued the chance to learn and grow together, recognizing individual strengths, celebrating progress, and reflecting back the best in one another. Through this process, confidence, leadership, patience, and care deepened, demonstrating that personal growth in this work is inseparable from collective growth and connection.

### Challenges identified by the YAAC

Engaging in new research required courage and the ability to navigate difficulties together. YAAC members spoke openly about difficulties that arose in research participation and design but consistently reframed obstacles as opportunities for learning and collaboration. Recognizing that everyone approaches challenges differently, they learned to trust each other’s process, knowing that this mutual faith helped move the group forward. Challenges became catalysts for adaptability and unity rather than setbacks.

Members also valued returning to earlier practices, such as lesson plans and team-building activities, which helped restore energy even on difficult days. Reflecting on the ups and downs, they emphasized patience and resilience – celebrating small victories and moments of connection that reminded them why the work matters. Even when outcomes were uncertain, they found satisfaction in seeing their efforts spark dialogue and awareness about gambling’s impact.

Creating a space where members could experiment, make mistakes, and express curiosity encouraged trust and collaboration grounded in care instead of hierarchy. Each obstacle brought greater understanding and underscored how growth often emerges through struggle.

## Conceptual and methodological considerations

### Positioning participatory action research as innovative in gambling harm research

Participatory action research (PAR) and its youth-focused form (YPAR) are widely used in public health to address complex behaviors and structural inequities but remain rare in gambling harm research. This study adapts YPAR to engage young adults as co-researchers in exploring gambling’s individual and community impacts, contributing an innovative example for the field.

Consistent with YPAR initiatives in substance use prevention (Weybright et al. 2024), YAAC members reported increased confidence, agency, and shared understanding through collaborative inquiry. YPAR’s effectiveness in engaging youth within their lived contexts (45–47) proved valuable here, as members highlighted gambling’s layered motivations and structural drivers. Their inquiry guiding question, “What is your why?,” framed gambling not as a single behavior but as experience shaped by intersection of personal, social and economic conditions.

Examples from other YPAR studies—such as youth-led studies on sexual and reproductive health and rights in Ontario (48) and gambling experiences in Malawi (20)—demonstrate how participatory research fosters skill-building, confidence, and creative dissemination. Similarly, ongoing community-based participatory initiatives in Springfield using photovoice (49) informed YAAC’s outreach and strengthened local relationships.

Together, these efforts underscore PAR’s unique potential in gambling harm research. By positioning young adults as co-researchers, this initiative expands participatory approaches into a field where youth perspectives have been largely absent. This centers lived experience and the identification and strength of collaborative knowledge, which lead to community-driven solutions.

### Implementing PAR with young adults

Implementing PAR with young adults involves logistical and structural challenges that shape collaboration. YAAC members balanced employment, education, caregiving, and housing and food insecurity while navigating systemic inequities such as racism and cisgenderism. Participation was therefore inseparable from the broader social conditions influencing daily life. Existing relationships with the partner organization (NNCC) fostered trust and continuity, enabling flexible engagement and access to wraparound supports. Time management was among the most persistent challenges: weekly evening meetings became insufficient as project demands increased, and remote work options were constrained by limited access to technology or quiet workspaces. A dedicated physical space for co-working might have improved participation and peer collaboration.

Funding limitations also affected sustainability. Although stipends honored participants’ time, reliance on short-term funding threatened long-term momentum. Transparent communication regarding financial constraints helped sustain trust, and achievements such as additional grants, conference presentations, and local partnerships reinforced engagement.

Balancing institutional expectations for measurable outcomes with community priorities of relationship-building required ongoing negotiation. Despite challenges that arose, YAAC members demonstrated enduring commitment—one YAAC member continued virtually after relocating—illustrating how shared purpose and relationships can transcend structural barriers. GARI’s experiences underscore the need for flexible, adequately resourced participatory frameworks that align with young adults’ lived realities. Equally, researchers and practitioners face logistical pressures in implementing PAR—balancing teaching, project management, and organizational responsibilities across limited time and funding. This project required significant coordination through frequent meetings, shared decision-making, and administrative support, highlighting the need to cost researcher time and institutional resources realistically. Additionally, participatory research demands compassion and an understanding that community needs may shift over time, even if that means adapting timelines or reconsidering predefined deliverables. This flexibility was not viewed as a failure to meet expectations, but as an intentional commitment to centering community priorities and trusting the process. In some cases, progress was reflected less in quantifiable outputs, such as the number of events held, and more in the strength of relationships and sustained engagement.

### Key lessons about collaboration across institutional partners

A key lesson learned was the importance of clearly defining partner roles from the outset. As the collaboration began, it became evident that establishing shared expectations early on would have supported more realistic planning around staffing, timelines, and deliverables. This reflection prompted greater intentionality in aligning resources with responsibilities and being transparent about what could reasonably be accomplished within the available time, staffing capacity, and competing demands.

Coordination across institutional partners also presented challenges. Although SDHHS, UMass, and NNCC shared a commitment to supporting the initiative and the YAAC, each operated within distinct systems, timelines, and accountability structures. From the university perspective, regulatory requirements, research activities, and publications took time but were important, even though they affected the overall timeline. From the health department perspective, institutional requirements such as grant funding accountability demanded multiple levels of approval. The need for stable timelines often conflicted with the iterative nature of participatory research. From the community organization’s perspective, its internal systems posed barriers, including challenges with stipend disbursement and project purchasing, which required creative workarounds, such as proactively adjusting timesheet submission timelines to avoid delayed pay due to holidays or payroll processing changes.

### Gambling as a public health issue

The topic of gambling itself posed unique challenges. Gambling is a highly political issue, particularly in a city that has supported casino development as a strategy to attract entertainment, increase revenue, and generate jobs, benefits that were projected to residents. The research team grappled with this tension between the desired community benefits and potential harms, recognizing that this complexity was compounded by pervasive gambling promotion toward young people, including the monetization of video games and constant exposure to messaging that emphasizes consumption and purchasing power. For young people with limited financial resources, these dynamics were often experienced as demoralizing reminders of economic instability within a broader capitalist system.

Radical love also called for ongoing attention to team care, recognizing that engaging deeply with stigmatized and politically sensitive topics can be emotionally taxing. A strength of this partnership was the range of expertise and resources available across organizational partners. With support from the Office of Problem Gambling within SDHHS and NNCC, the team was well-positioned to provide support if and when needed. This included access to gambling-specific resources as well as broader services such as food and housing program assistance. As a result, YAAC members express appreciation for the support provided by all partners. Furthermore, several practices helped tend to members’ well-being throughout the project. For example, the team co-created community guidelines at the outset, establishing shared agreements about what would help everyone feel comfortable and respected. Regular check-ins made space for members to share how they were doing more broadly, not just in relation to the work. Community-building and mutual aid were woven into the culture of the work. When someone was having a hard time, the group made room for that. This sometimes looked like meditative exercises, a walk together, or a group picnic. These moments reflected radical love in action: seeing team members as full people, not just contributors to a project, and showing up for one another accordingly.

Conducting research on gambling among young adults revealed nuanced and varied experiences—some direct, others shaped by family, peers, or community norms. Much time was spent unpacking these layers, identifying hidden behaviors not always recognized as gambling or viewed as problematic. Group discussions highlighted how games such as scratch tickets and online loot boxes are culturally normalized, showing the need for a broader definition of gambling in community contexts. Similar to findings reported among young adults in the UK (50), GARI YAAC members described the pervasiveness of gambling advertisements across multiple platforms—including in-person spaces, television, and social media—as well as the relative ease of bypassing age-verification systems intended to restrict access. They also expressed support for stronger regulatory reform to address these concerns. Lastly, the YAAC consistently reflected on how the challenges associated with emerging adulthood, alongside ongoing mental health stressors, may contribute to engagement in gambling. They described how gambling-related harms can then create reinforcing cycles that further exacerbate financial, emotional, and relational challenges. These observations align with findings from existing research on gambling harms (51).

Approaches to addressing gambling from a public health perspective sometimes created tension between harm-reduction models promoted by the funder and prevention-based perspectives emphasized by leading public health institutions. The team navigated competing narratives and experiences, including messages around what constituted “responsible gambling,” while acknowledging that such language can feel misaligned when addiction and structural inequities are at play.

### Evaluation and feasibility

During Phase 1, feasibility was assessed through the development and outcomes of the CAB (later renamed the YAAC by its members). Key indicators included the ability to recruit young adults and sustain ongoing participation over the six-week meeting period. Attendance was strong, and CAB/YAAC members were able to engage in substantive discussions about gambling and gambling-related harms. A central outcome of this phase was the identification of thematic areas that reflected issues young adults viewed as most relevant to gambling in their lives and communities. The identification of these themes and CAB/YAAC members’ interest in continuing the work demonstrated readiness to move into the next phase.

Phase 2 focused on the development of a PAR plan. Feasibility at this stage was assessed by whether the group could collectively translate identified priorities into a concrete research plan that YAAC members could take ownership of and realistically implement with sustained engagement. The development of a PAR plan, driven by YAAC input and aligned with available resources, served as the primary marker of progress and feasibility. While attendance varied during this time, the group met regularly over 8 months, and by the end, four YAAC members remained firmly committed to continuing the PAR plan.

Phase 3 centered on implementing the PAR plan and understanding whether YAAC members could feasibly co-lead and carry out the proposed activities. This assessment occurred at both the overall coordination level and within the two research components: digital storytelling and the creative crowdsourcing contest. The digital storytelling method had its challenges, e.g., competing schedules and varied availability required flexibility, adjusting timelines, offering one-on-one support, and finding creative ways to sustain engagement. As a result, this part of the research took longer than anticipated. Some of the YAAC with less direct gambling experience needed additional time to conceptualize their perspective on gambling harm experienced on a socioeconomic and community level.

A creative crowdsourcing contest further tested outreach strategies. Limited public dialogue on gambling, especially among youth and young adults, coupled with stigma, made it difficult to generate enthusiasm (52–54). While arts-based approaches can powerfully foster expression, some members of the GARI team who live in Springfield felt that there is an emerging arts scene that offered limited infrastructure compared with nearby communities. Also, outreach emerged as particularly resource intensive. While YAAC members engaged in event-based outreach, broader street-level outreach was more challenging due to limited availability, reliance on public transportation, and competing work schedules, especially during daytime hours. Outreach efforts were more effective when supported by the SDHHS lead, which provided access to a vehicle and enabled travel to targeted locations that would otherwise have been difficult to reach. Weekend events also proved more feasible for engagement, as they aligned better with YAAC members’ availability. YAAC members view these hurdles as valuable learning experiences that deepened their understanding of effective community engagement. Reaching beyond immediate networks was particularly difficult within the city’s geographic boundaries, yet it revealed genuine interest from neighboring areas and opportunities for future expansion. Despite constraints in design and context, the group remains optimistic—seeing each challenge as a lesson for broadening participation and imagining new ways to extend the reach and impact of this work.

Across phases, feasibility decisions were guided by ongoing reflection and reassessment as partners. Progress was assessed through regular check-ins among all partners, attention to participation patterns, and the collective sense of whether the work remained manageable, meaningful, and aligned with project goals. When challenges arose, such as varied engagement or logistical constraints, adaptations were made to timelines, outreach strategies, and support.

## Conclusion

GARI offers an example of what it looks like to practice public health research through shared inquiry, relationship-building, and collective growth. By partnering with young people as co-researchers and co-leaders, the project not only reimagined who holds knowledge but also how that knowledge is created, interpreted, and implemented in research. Through its emphasis on arts-based and participatory approaches, GARI contributes to the expanding body of public health scholarship that calls for more community-centered and contextually grounded ways to understand issues such as gambling harms.

The process of developing and implementing this work has underscored the power and complexity of participatory approaches to research. It has highlighted how equity, transparency, and relational trust must be continually cultivated as daily practices that guide every decision and interaction. As the public health field continues to confront complex health and social issues, GARI calls for broader adoption of community-driven, arts-based methodologies that invite people to be active participants in shaping both the questions and the pathways toward change. Such approaches hold the potential to deepen understanding, challenge extractive research norms, and strengthen the connections between the community and academia.

## Data Availability

The original contributions presented in the study are included in the article/supplementary material, further inquiries can be directed to the corresponding author.

## References

[1] Calado F Griffiths MD. Problem gambling worldwide: an update and systematic review of empirical research (2000–2015). J Behav Addict. (2016) 5:592–613. Located at: edscee.619862. doi: 10.1556/2006.5.2016.073, 27784180 PMC5370365

[2] Gainsbury S Russell A Hing N Wood R Lubman D Blaszczynski A. How the internet is changing gambling: findings from an Australian prevalence survey. J Gambl Stud. (2015) 31, 31:1–15. doi: 10.1007/s10899-013-9404-7, 23934369 PMC4611023

[3] Wardle H Degenhardt L Marionneau V Reith G Livingstone C Sparrow M . The lancet public health commission on gambling. Lancet Public Health. (2024) 9:e950–94. doi: 10.1016/S2468-2667(24)00167-1 PubMed 39491880, 39491880

[4] Volberg RA Williams RJ Zorn M Evans V. Gambling and Problem Gambling in Massachusetts: Results of a Follow-up Population Survey. Amherst, MA: School of Public Health and Health Sciences, University of Massachusetts Amherst (2023) (Social and Economic Impacts of Gambling in Massachusetts (SEIGMA) Project). Report No.

[5] Hing N Russell A Tolchard B Nower L. Risk factors for gambling problems: an analysis by gender. J Gambl Stud. (2016) 32:511–34. Located at: 2016-25630-010. doi: 10.1007/s10899-015-9548-8, 25948418 PMC4875054

[6] United States Census Bureau. American community survey (ACS) 2019–2023 5-year estimates: Springfield city, Massachusetts [internet]. (2023). Available online at: https://data.census.gov

[7] Volberg RA Zorn M Evans V Stanek EJ Williams RJ. Impact of MGM Springfield on Gambling Attitudes, Participation and Problem Gambling [Internet]. Amherst, MA: School of Public Health and Health Sciences, University of Massachusetts Amherst (2020) (Social and Economic Impacts of Gambling in Massachusetts (SEIGMA) Project). Report No.

[8] Feeny D. National Survey on Gambling Attitudes and Gambling Experiences 2.0 [Internet].National Council on Problem Gambling (2023).

[9] Aymamí N Jiménez-Murcia S Granero R Ramos-Quiroga JA Fernández-Aranda F Claes L . Clinical, psychopathological, and personality characteristics associated with ADHD among individuals seeking treatment for gambling disorder. Biomed Res Int. (2015) 2015:965303. doi: 10.1155/2015/965303, 26229967 PMC4502275

[10] Lynch WJ Maciejewski PK Potenza MN. Psychiatric correlates of gambling in adolescents and young adultsgrouped by age at gambling onset. Arch Gen Psychiatry. (2004) 61:1116–22. doi: 10.1001/archpsyc.61.11.1116, 15520359

[11] Zendle D Meyer R Over H. Adolescents and loot boxes: links with problem gambling and motivations for purchase. R Soc Open Sci. (2019) 6:190049. doi: 10.1098/rsos.190049, 31312481 PMC6599795

[12] Russell AMT Hing N Browne M Li E Vitartas P. Who bets on Micro events (microbets) in sports? J Gambl Stud. (2019) 35:205–23. doi: 10.1007/s10899-018-9810-y, 30386964

[13] Mazar A Williams RJ Stanek EJ Zorn M Volberg RA. The importance of friends and family to recreational gambling, at-risk gambling, and problem gambling. BMC Public Health. (2018) 18:1080. doi: 10.1186/s12889-018-5988-2, 30165837 PMC6117965

[14] Zhao Y Marchica L Derevensky JL Ivoska W. Mobile gambling among youth: a warning sign for problem gambling? J Gambl Issues. (2018) 38:268–82. doi: 10.4309/jgi.2018.38.14

[15] Leavy P. Handbook of Arts-Based Research. New York, NY: The Guilford Press (2018).

[16] Gubrium A Fiddian-Green A Lowe S DiFulvio G Peterson J. Digital storytelling as critical narrative intervention with adolescent women of Puerto Rican descent. Crit Public Health. (2019) 29:290–301. doi: 10.1080/09581596.2018.1451622, 31130780 PMC6531859

[17] Gubrium AC Fiddian-Green A Lowe S DiFulvio G Del Toro-Mejías L. Measuring down: evaluating digital storytelling as a process for narrative health promotion. Qual Health Res. (2016) 26:1787–801. doi: 10.1177/1049732316649353, 27184518

[18] Lohr AM Raygoza Tapia JP Valdez ES Hassett LC Gubrium AC Fiddian-Green A . The use of digital stories as a health promotion intervention: a scoping review. BMC Public Health. (2022) 22:1180. doi: 10.1186/s12889-022-13595-x, 35698097 PMC9192132

[19] Gorry E Salerno Valdez E Chan J Dixon S Davidson Carroll G Phuntsog T . On caring and a love ethic to address the effects of structural violence on adolescent health. Child Youth Serv Rev. (2024) 163:107760. doi: 10.1016/j.childyouth.2024.107760

[20] Mtema O Singano I‘S McGee D Yakobe Y Sichali J Makamo M . ‘Creating poverty chances’: young people confront gambling harms in Malawi. Sociol Res Online. (2024) 29:1089–96. doi: 10.1177/13607804231207152

[21] Fox M Fine M. Leadership in solidarity: notions of leadership through critical participatory action research with young people and adults. New Dir Stud Leadersh. (2015) 148:45–58. doi: 10.1002/yd.20152, 26895168

[22] Fakoya I Cole C Larkin C Punton M Brown E Ballonoff SA. Enhancing human-centered design with youth-led participatory action research approaches for adolescent sexual and reproductive health programming. Health Promot Pract. (2022) 23:25–31. doi: 10.1177/15248399211003544, 33858267

[23] hooks bell. Feminist Theory: From Margin to Center. 2nd ed. Cambridge, MA: South End Press (2000).

[24] Feekery A. The 7 c’s framework for participatory action research: inducting novice participant-researchers. Educ Action Res. (2024) 32:332–47. doi: 10.1080/09650792.2023.2234417

[25] Bődi CB Amendola A Bright MA. Community-based participatory research: involving young people with lived experiences of problematic sexual behaviors. Prev Sci. (2025) 26:814–26. doi: 10.1007/s11121-025-01816-9, 40461877

[26] Marrone N Nieman C Coco L. Community-based participatory research and human-centered design principles to advance hearing health equity. Ear Hear. (2022) 43:33S–44S. doi: 10.1097/AUD.0000000000001183, 35724253 PMC9219558

[27] Israel BA Schulz AJ Coombe CM Parker EA Reyes AG Rowe Z . "Community-based participatory research: an approach to research in the urban context". In: Galea S Ettman CK Vlahov D, editors. Urban Health [Internet]. New York (online edn): Oxford University Press (2019). p. 272–82.

[28] Israel BA Schulz AJ Parker EA Becker AB. Review of community-based research: assessing partnership approaches to improve public health. Annu Rev Public Health. (1998) 19:173–202. doi: 10.1146/annurev.publhealth.19.1.173, 9611617

[29] Minkler M Wallerstein N. Community-Based Participatory Research for Health: From Process to Outcomes. 2nd ed. San Francisco, CA: Jossey-Bass (2008).

[30] brown adrienne maree. Holding Change: The Way of Emergent Strategy Facilitation and Mediation. Chico, CA: AK Press (2021).

[31] Lambert J Hessler B. Digital Storytelling: Story work for urgent times. 6th ed.Digital Diner Press (2020). Available online at: https://www.storycenter.org/inventory/p/digital-storytelling-story-work-for-urgent-times

[32] Kendrick C MacEntee K Flicker S. Exploring audience engagement and critical narrative intervention with the celling sex film. Health Promot Pract. (2021):33S–43S. doi: 10.1177/15248399211040492, 34664517 PMC8739580

[33] Flicker S Wilson C Monchalin R Oliver V Prentice T Jackson R . “Stay strong, stay sexy, stay native”: storying indigenous youth HIV prevention activism. Action Res. (2019) 17:323–43. doi: 10.1177/1476750317721302

[34] Valdez E Chan J Dixon S Carroll GD Phuntsog T Delorme E . Participatory action research to explore the role of structural violence on marginalized and racialized young parents. Health Educ Behav. (2024) 51:229–39. doi: 10.1177/10901981231197397, 37746721

[35] Brabham DC Ribisl KM Kirchner TR Bernhardt JM. Crowdsourcing applications for public health. Am J Prev Med. (2014) 46:179–87. doi: 10.1016/j.amepre.2013.10.016 24439353

[36] Tucker JD Day S Tang W Bayus B. Crowdsourcing in medical research: concepts and applications. PeerJ. (2019) 7:e6762. doi: 10.7717/peerj.6762, 30997295 PMC6463854

[37] White JJ Mathews A Henry MP Moran MB Page KR Latkin CA . A crowdsourcing open contest to design pre-exposure prophylaxis promotion messages: protocol for an exploratory mixed methods study. JMIR Res Protoc. (2020) 9:e15590. doi: 10.2196/15590, 31899456 PMC6969383

[38] Morton S Tuff R Beckwith K Banks M Dixon-Terry E. Children’s poster contest on healthy eating. Californian J Health Promot. (2005) 3:70–2. doi: 10.32398/cjhp.v3i1.1743

[39] Evans LA Gomez O Jiménez DJ Williamson HJ Carver AT Parthasarathy S . Engaging youth and young adults in the COVID-19 pandemic response via the “it’s our turn” crowdsourcing contest: international journal of environmental research and public health. Int J Environ Res Public Health. (2023) 20:5112. doi: 10.3390/ijerph20065112, 36982019 PMC10049566

[40] Mathews A Conserve D Mason H Alston LM Rennie S Tucker J. ‘Informed and empowered’: a mixed-methods study of crowdsourcing contests to promote uptake of HIV self-testing kits among African Americans. J Virus Erad. (2020) 6:74–80. doi: 10.1016/S2055-6640(20)30020-0, 32405425 PMC7213069

[41] Hayman LW Jr. Radical love as a public health initiative. The Center for Contemplative Mind in society 3rd annual Arthur Zajonc lecture on contemplative education. (2019).

[42] Transforming Communities: Technical Assistance, Training & Resource Center. Building Beloved Community. The Challenges and Opportunities of Mobilizing and Organizing Communities to Prevent and Respond to Domestic Violence [Internet]. Transforming Communities: Technical Assistance, Training & Resource Center (2014).

[43] Miles S Bright L Burgess P DeCambra MH Enos RK Kalilihiwa G . Building the beloved community: reflections in understanding relationships to food for native Hawaiians. Prog Community Health Partnersh. (2018) 12:483–7. doi: 10.1353/cpr.2018.0073, 30739902

[44] Warren RC Walker BJ Maclin SDJ Miles-Richardson S Tarver W James CM. Respecting and protecting the beloved community, especially susceptible and vulnerable populations. J Health Care Poor Underserved. (2011) 22:3–13. doi: 10.1353/hpu.2011.0104, 21857132

[45] Helm S Lee W Hanakahi V Gleason K McCarthy K. Using PHOTOVOICE with youth to develop a drug prevention program in a rural HAWAIIAN community. Am Indian Alsk Native Ment Health Res Online. (2015) 22:1–26. doi: 10.5820/aian.2201.2015.1, 25768388 PMC4401743

[46] Valdez ES Valdez L Garcia DO. Using participatory methods to enhance youth engagement in substance use research. Health Promot Pract. (2021) 22:747–9. doi: 10.1177/1524839921990005, 33611963 PMC8377079

[47] Linton SL Winiker A Tormohlen KN Schneider KE McLain G Sherman SG . “People don’t just start shooting heroin on their 18th birthday”: a qualitative study of community stakeholders’ perspectives on adolescent opioid use and opportunities for intervention in Baltimore, Maryland. Prev Sci. (2021) 22:621–32. doi: 10.1007/s11121-021-01226-7, 33826057 PMC8024438

[48] Vandermorris A Wigle J Tam M Peresin J Dalal S Kwong I . Application of youth-led participatory action research to examining adolescent sexual and reproductive health and rights in Ontario: what can we learn? Health Promot Pract. (2025) 26:913–25. doi: 10.1177/15248399241298836, 39691996 PMC12332221

[49] Massachusetts Photovoice Project. Problem gambling prevention [internet]. Available online at: https://mcoepgp.org/prevention-approaches/massachusetts-photovoice-project/

[50] Torrance J Roderique-Davies G Thomas SL Davies N John B. ‘It’s basically everywhere’: young adults’ perceptions of gambling advertising in the UK. Health Promot Int. (2021) 36:976–88. doi: 10.1093/heapro/daaa126, 33270845

[51] Gavriel-Fried B Malka I Levin Y. The dual burden of emerging adulthood: assessing gambling severity, gambling-related harm, and mental health challenges. Int J Environ Res Public Health. (2024) 21:702. doi: 10.3390/ijerph21060702, 38928948 PMC11203917

[52] Quigley L Prentice J Warren JT Quilty LC Dobson KS Hodgins DC. What’s in a name? Evaluating the public stigma of gambling disorder. J Gambl Stud. (2020) 36:1205–28. doi: 10.1007/s10899-019-09924-2, 31848837

[53] van Baal ST Bogdanski P Daryanani A Walasek L Newall P. The lived experience of gambling-related harm in natural language. Psychol Addict Behav. (2025) 39:397–409. doi: 10.1037/adb0001030, 39250242

[54] Wöhr A Wuketich M. Perception of gamblers: a systematic review. J Gambl Stud. (2021) 37:795–816. doi: 10.1007/s10899-020-09997-4, 33660191 PMC8364520

