# Correction: Place your bet on change: the Young Adult Action Collective leading community-academic partnership efforts to understand and address gambling harms in Springfield, MA

**DOI:** 10.3389/fpubh.2026.1904828

**Published:** 2026-06-23

**Authors:** Frontiers Production Office

**Affiliations:** Frontiers Media SA, Lausanne, Switzerland

**Keywords:** crowdsourcing contest, digital storytelling, gambling harm, participatory action research, young adults

In the **Generative AI statement**, it erroneously stated that the authors used Generative AI and the Alt-Text for Figure 1 was mistakenly inserted in the statement too.

The correct **Generative AI statement** is “The author(s) declared that no Generative AI was used in the creation of this manuscript.

Any alternative text (alt text) provided alongside figures in this article has been generated by Frontiers with the support of artificial intelligence and reasonable efforts have been made to ensure accuracy, including review by the authors wherever possible. If you identify any issues, please contact us.”

The original version of this article has been updated.

